# No association between different in tibial resection level and clinical outcomes six months after Oxford unicompartmental knee arthroplasty: a prospective cohort study

**DOI:** 10.1186/s12891-022-06088-w

**Published:** 2023-01-03

**Authors:** Chaturong Pornrattanamaneewong, Naruepol Ruangsillapanan, Pakpoom Ruangsomboon, Rapeepat Narkbunnam, Keerati Chareancholvanich, Pacharapol Udomkiat

**Affiliations:** 1 Department of Orthopaedic Surgery, Faculty of Medicine Siriraj Hospital, Mahidol University, 2 Wang Lang Road, Bangkok, Bangkok Noi 10700 Thailand; 2 Department of Orthopaedic Surgery, Maharat nakornratchasima hospital, Nakhon Ratchasima, Thailand; 3 Institute of Health Policy, Management and Evaluation, University of Toronto, Toronto, Canada

**Keywords:** Investigation, Association, Depth, Tibial resection, Medial knee pain, Oxford unicompartmental knee arthroplasty

## Abstract

**Background:**

Localized tibial strain is one of the hypotheses to explain residual pain after Oxford UKA. We evaluate whether the depth of the vertical cut during tibial resection correlates with medial knee pain. We aimed to investigate the association between the depth of tibial resection and medial knee pain after OUKA.

**Methods:**

This prospective cohort study enrolled 85 patients (mean age: 64.5 ± 7.7 years) who underwent cemented OUKA at our institute during October 2018–June 2019. The depth of tibial resection was measured intraoperatively as the thickness of the anterior, middle, and posterior parts. The greatest of the three thicknesses was recorded. Medial knee pain was assessed at 6 weeks and followed to 6 months. Patients were divided into the pain (P) and no pain (NP) groups. Preoperative and postoperative radiographic findings and OKS were compared between groups. We used logistic regression to analyze the independent association.

**Results:**

The mean preoperative Oxford Knee Score (OKS) was 27.2 ± 7.6. The incidence of medial knee pain was 23.5% at 6 weeks after OUKA. The P group had a significantly lower OKS at 6 weeks compared to the NP group (28.9 ± 9.7 vs 33.7 ± 6.5, *p* = 0.049). There was no significant difference in the depth of tibial resection between groups. Medial knee pain had resolved by 6 months in all patients, and the 6-month OKS was similar between groups.

**Conclusion:**

Medial knee pain was found to be common in the early postoperative period after OUKA, but this pain spontaneously resolved by 6 months. As a range of tibial resection level, post-operative pain is not associated with tibial resection thickness in this study.

**Level of evidence:**

Level II.

**Trial registration:**

The study was approved by the Institutional review board of Siriraj Hospital, Mahidol university. [SIRB 691/2560(EC4)].

## Introduction

Mobile-bearing unicompartmental knee arthroplasty (UKA) is an effective treatment for medial compartmental osteoarthritis (OA). It provides good clinical results and long-term survivorship [1]. However, several national registries have reported a 10-year survival rate of 81–88% [2, 3], which is lower than that of total knee arthroplasty (TKA). To understand the modes of failure, van der List, et al. [4] conducted a systematic review to assess the medial UKA and found the three most common causes of failure to be aseptic loosening (35%), OA progression (24%), and pain (14%).

Medial knee pain is a common complaint that leads to unsatisfactory outcomes after mobile-bearing UKA. The incidence of this problem was reported to widely range from 1 to 55% [5, 6]. Several possible causes have been proposed, including impingement, overhanging of the tibial component, cementing errors, loosening of the prosthesis, neuroma formation, and medial soft tissue irritation, such as irritation of the medial collateral ligament and/or pes anserinus [6]. Localized tibial strain has also been hypothesized as a potential cause of this pain. Finite element studies conducted by Pegg, et al. [7] revealed increased tibial strain after UKA. Other studies found that coronal malalignment of the tibial component exerted an effect on the medial proximal tibial bone after UKA [8, 9]. A recent systematic review of 15 studies reported excellent survivorship of OUKA, with a rate of 93% at 10 years and 89% at 15 years. Unexplained knee pain was the fourth most common reason for revision with an incidence of 0.57% [10]. Defining the success of OUKA, most studies reported using functional outcomes, such as OKS [6], and Hospital for Special Surgery Knee Score [11], and implant survivorship [5, 10, 12]. Data specific to medial knee pain in this setting remains scarce.

Concerning bone resection depth, Berend, et al. [13] reported that greater resection depth increased strain at the proximal tibia; however, those authors conducted their biomechanical study in TKA model. The present study is the first to investigate the association between the depth of tibial resection and medial knee pain after Oxford unicompartmental knee arthroplasty (OUKA).

## Materials and methods

This prospective cohort study consecutively enrolled patients who underwent cemented OUKA at our institute from October 2018 to June 2019. All included patients were diagnosed with an anteromedial OA (Kellgren-Lawrence grade 3 or 4). The indications for OUKA were varus deformity < 15°, flexion contracture < 15°, and knee flexion at least 110°. Patients with psychological problems, previous knee surgery, significant hip or spine problem, or follow-up time < 6 months were excluded. This study was approved by our center’s institutional review board (IRB), and written informed consent was obtains from all study patients.

All procedures were performed by a single experienced surgeon (PU). After inflating the tourniquet to a pressure of 300 mmHg, the mini-medial parapatellar approach was used [14]. The final decision to perform UKA was made intraoperatively by checking the following conditions: no severe damage to the lateral part of the patellofemoral joint, such as bone loss, grooving, or subluxation; intact functionally of both cruciate ligaments; and a preserved lateral compartment with intact lateral meniscus and full-thickness articular cartilage. Oxford partial knee micro-plasty instrumentation was used to perform OUKA without patellar dislocation. After the removal of all osteophytes, the front of the tibia was exposed without releasing any fibers of the medial collateral ligament. Tibial plateau resection was performed using a femoral sizing spoon, tibial saw guide with standard 0 mm tibial shim, and 3 mm G-clamp in all knees. The direction of the tibial cut was aimed perpendicular to the mechanical axis with 5–7 degrees of the posterior slope. The vertical saw cut was passed through the edge of the anterior cruciate ligament insertion and pointed parallel to the sagittal axis of the tibia. Then we followed the remaining surgical steps, according to the manufacturer’s surgical technique guide. The cemented Oxford phase 3, mobile-bearing UKA was implanted (Zimmer Biomet, Warsaw, Indiana, USA). The posterior-medial side is a difficult-to-visualize critical area during surgery. Therefore, it is essential to carefully match the resected tibial bone fragment to the contralateral tibial size template in order to reduce the likelihood of posteromedial protusion. Medial overhanging (> 1 mm) of the femoral component and anteromedial overhanging (> 1 mm) of the tibial component were easily detected and avoided by visual inspection. We applied only a moderate amount of cement on the tibial surface to avoid cementophyte. Likewise, we thoroughly cleared excessive cement at the medial and posterior aspects of the tibial tray multiple times after compressing a component into place. No cementing error at the medial side was detected in our series.

The postoperative rehabilitation protocol was started as soon as possible, including ankle pumping, range-of-motion exercise, and out-of-bed ambulation. Cold compression was applied for at least 8 hours per day. All patients received the same postoperative pain management protocol. No intraoperative or postoperative complications were observed in this study.

**Fig. 1:**
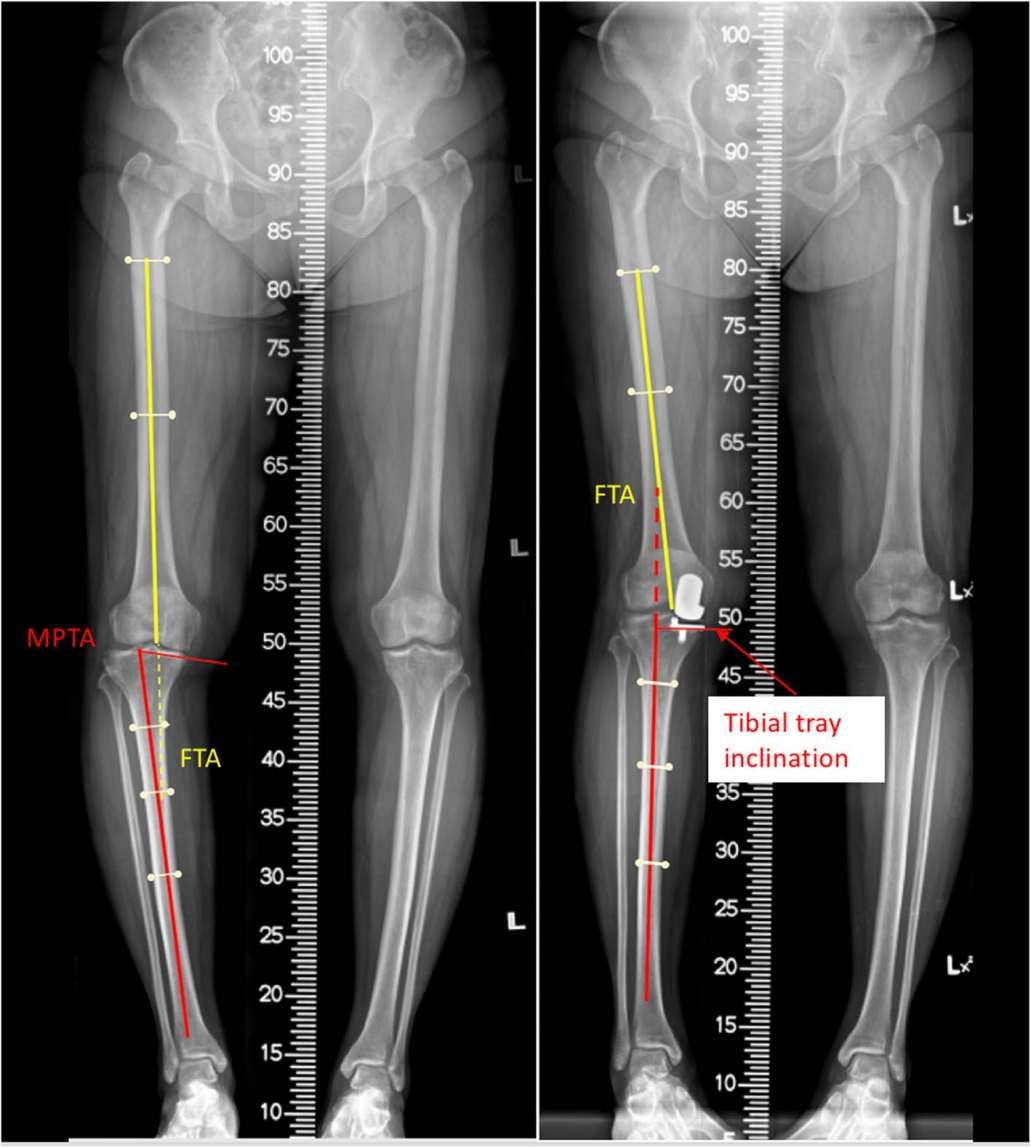
Mechanical femorotibial angle (FTA) and medial proximal tibial angle (MPTA), and tibial tray inclination were measured similarly. (Left) preoperative and (Right) postoperative radiographic measurement

**Fig. 2:**
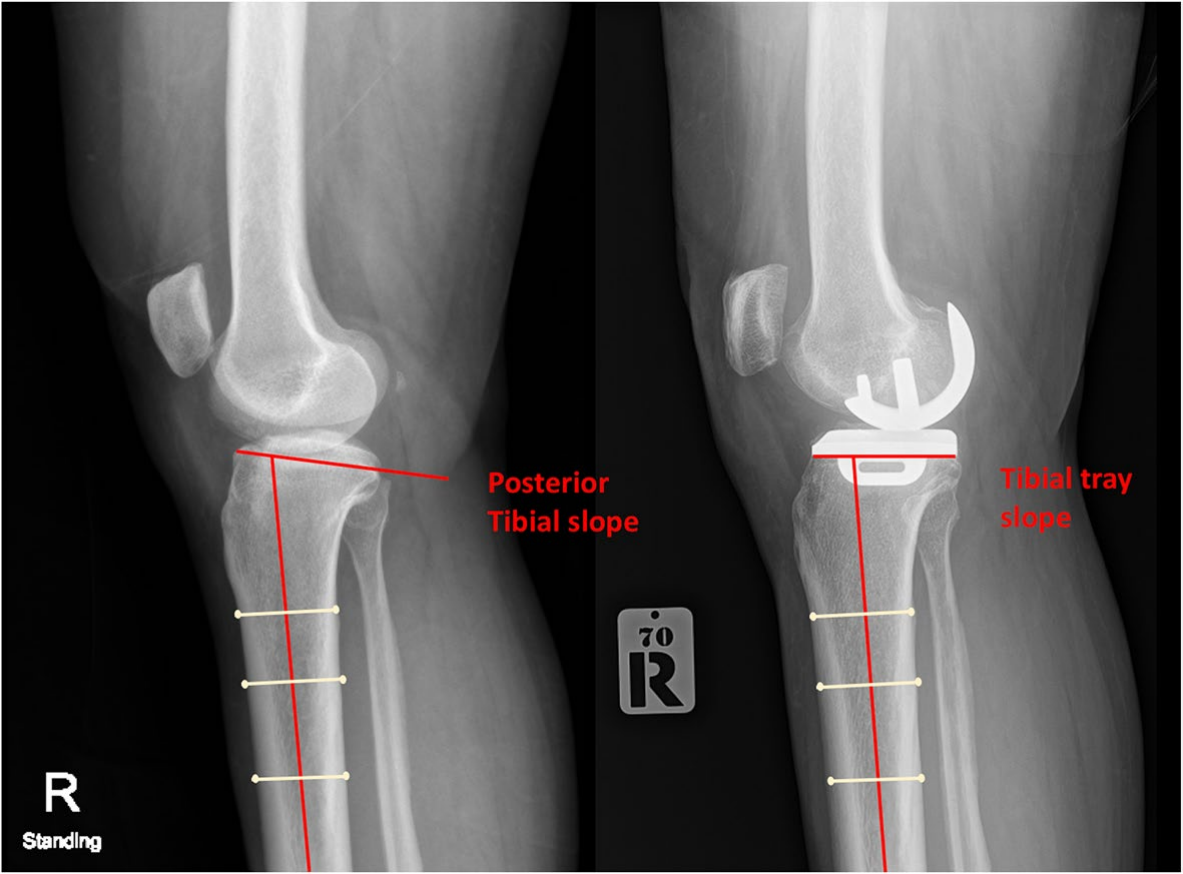
The posterior tibial slope was the angle formed between the line perpendicular to the sagittal tibial axis and the line passing through the tibial plateau’s highest anterior and posterior points. The tibial tray slope was the angle formed between the line perpendicular to the sagittal tibial axis and the tibial tray surface

Demographic and clinical data were collected, including age, gender, body weight, height, body mass index (BMI), and preoperative Oxford knee score (OKS). Anteroposterior hip-knee-ankle (HKA) radiographs were evaluated preoperatively and at 4 weeks after surgery. Mechanical femorotibial angle (FTA) was measured in both radiographs. Medial proximal tibial angle (MPTA) and tibial tray inclination were measured by a similar method from preoperative and postoperative HKA radiographs, respectively (Fig. 1). Preoperative posterior tibial slope and postoperative tibial tray slope were measured on lateral knee radiographs according to the method published by Kizilgoz, et al. [15]. The longitudinal sagittal axis of the tibia was drawn as a line passing through two points located in the center of the anteroposterior width of the tibia at 6 and 10 cm apart on the tibial plateau or tibial tray surface. The posterior tibial slope was the angle formed between the line perpendicular to the sagittal tibial axis and the line passing through the highest anterior and posterior points of the tibial plateau, whereas the tibial tray slope was the angle formed between the line perpendicular to the sagittal tibial axis and the tibial tray surface (Fig. 2).

**Fig. 3:**
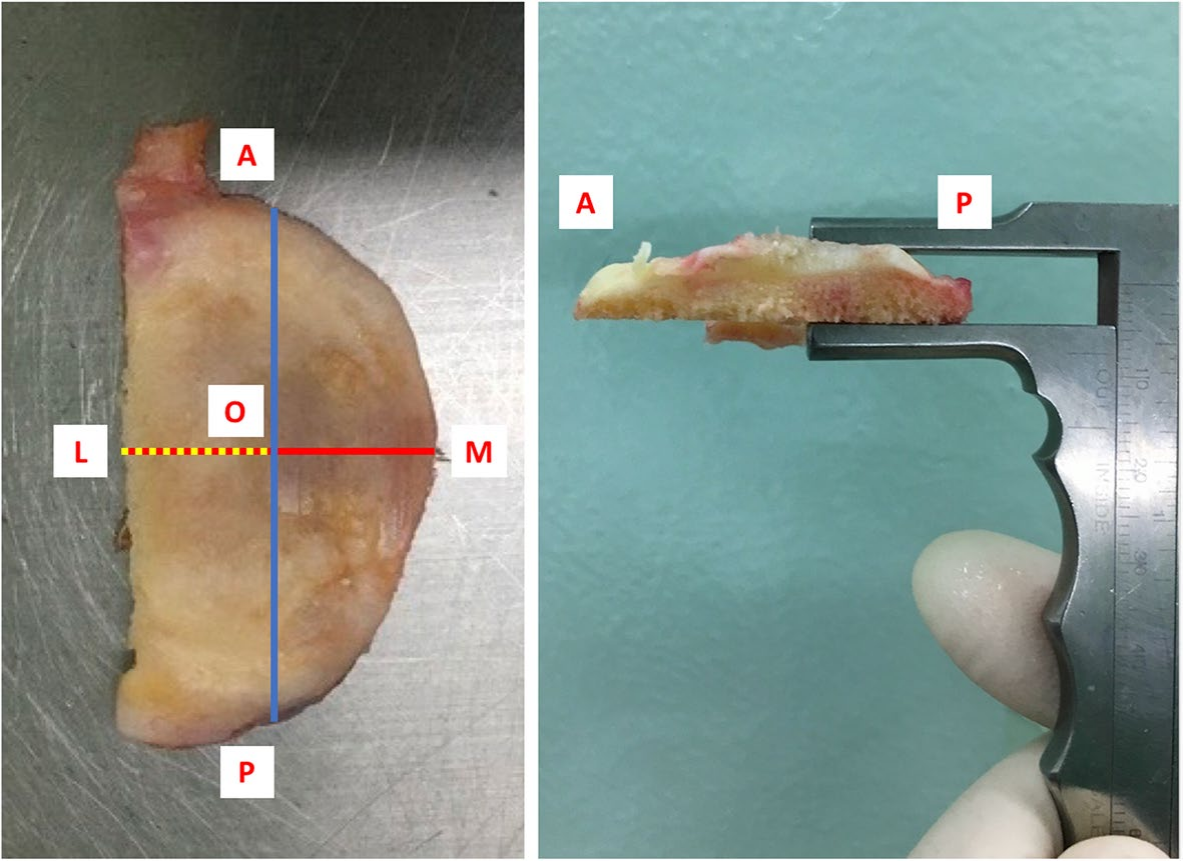
The depth of tibial resection was measured intraoperatively using a Vernier caliper. The maximal depth was determined as the maximal thickness among the three parts in each patient

The depth of tibial resection was measured intraoperatively using a Vernier caliper according to the following method. First, the widest part of the resected fragment perpendicular to the lateral border was identified using the medial-lateral (ML) line. The midpoint of the ML line was marked and defined as the O point. The anteroposterior (AP) line was drawn perpendicular to the ML line and then passed through the O point. Lastly, the measurement of tibial resection depth was recorded in three parts, as follows: 1) the anterior part was the maximal thickness on the AO line, 2) the middle part was the maximal thickness on the MO line, and 3) the posterior part was the maximal thickness on the OP line (Fig. 3). The maximal depth was determined as the maximal thickness among the three parts in each patient. The size of the prosthesis and thickness of the polyethylene was also recorded.

Medial knee pain and OKS were collected at both 6 weeks and 6 months after OUKA.

Medial knee pain was defined as pain at the anteromedial area of the proximal tibia in the absence of any of the following signs: 1) sign of inflammation (swelling, warmth, and erythema), 2) positive Tinel’s sign, and 3) increasing pain on knee motion or during resisted knee flexion. The anteromedial area was defined as the area within the following boundaries: the medial joint line, the medial border of the tibial tubercle, the lowest part of the tibial tubercle, and the midpoint of the most anterior and most posterior parts of the knee. Complications, including venous thromboembolism, fracture, and infection, were also recorded during the follow-up period.

### Sample size calculation and statistical analysis

Our sample size for this study was calculated using data from a study by Edmonson, et al. [6]. They reported a poor correlation (correlation coefficient < 0.3) between OKS and radiographic score after UKA. Using their data, 80% power, and an alpha error of 0.05, a sample size of 85 knees was calculated. We then decided to increase the sample size to 95 patients to compensate for a potential 10% dropout rate.

Continuous data are presented as mean ± standard deviation, and categorical data are given as numbers and percentages. According to the presence of medial knee pain at 6 weeks, the cohort was divided into the pain (P) and no pain (NP) groups. Independent *t*-test, chi-square test, or Fisher’s exact test was used to compare variables between groups depending on the distribution of data. Identification of factors independently associated with medial knee pain and variables with *p*-value < 0.2 were entered into the logistic regression model. Univariate and multivariate analyses were then performed. A *p*-value < 0.05 was regarded as being statistically significant. All data analyses were performed using SPSS Statistics software (SPSS, Inc., Chicago, IL, USA).

## Results

A total of 95 knees were recruited. Of those, 10 knees were excluded due to anterior cruciate ligament (ACL) insufficiency (4 knees), lateral compartment damage (3 knees), and loss to follow-up (3 knees). The remaining 85 knees (mean age of patients: 64.5 ± 7.7 years) were included in the final analysis. The patient demographic and clinical characteristics of this cohort are shown in Table 1. At postoperative week 6, 20 patients (23.5%) reported medial knee pain. There were no significant differences in preoperative data between the P and NP groups (Table 1).

**Table 1:** Patients’ characteristics

Characteristics	Overall (*n* = 85)	No pain (*n* = 65)	Pain (*n* = 20)	*p*-value
Age (yr)	64.5 ± 7.7	64.4 ± 8.0	64.7 ± 6.9	0.880
Gender (female,%)	73 (85.9%)	57 (87.7%)	16 (80%)	0.464
Side (right,%)	46 (54.1%)	36 (55.4%)	10 (50.0%)	0.673
Body weight (kg)	68.3 ± 11.6	69.1 ± 11.8	65.5 ± 10.9	0.226
Height (cm)	156.7 ± 7.3	156.5 ± 7.9	157.2 ± 5.1	0.716
BMI (kg/m^2^)	27.8 ± 4.3	28.2 ± 4.2	26.6 ± 4.6	0.147
Preoperative OKS (score)	27.2 ± 7.6	27.9 ± 7.4	25.1 ± 8.3	0.146
Preoperative radiographic findings				
FTA (°)	173.3 ± 2.5	173.4 ± 2.7	173.0 ± 1.9	0.464
MPTA (°)	86.3 ± 2.2	88.4 ± 2.3	86.2 ± 1.9	0.747
Tibial slope (°)	8.3 ± 2.7	8.3 ± 2.6	8.5 ± 3.1	0.753

*BMI* body mass index, *OKS* Oxford knee score, *FTA* mechanical femorotibial angle, *MPTA* medial proximal tibial angle

Intraoperatively, the mean depths of tibial resection at the anterior, middle and posterior parts were 7.3 ± 1.9, 4.9 ± 1.8, and 5.1 ± 1.7 mm, respectively. There was no significant difference in all parts or the maximum of tibial depth resection between groups. The significant difference in tibial component size was observed between groups (*p* = 0.037) (Table 2). Regarding postoperative radiographic evaluation, there was no significant difference in FTA (*p* = 1.000) or tibial inclination (*p* = 0.589) between groups. However, the P group had a significantly lower OKS at 6 weeks compared to the NP group (*p* = 0.049). Medial knee pain spontaneously resolved in all study patients by 6 months after surgery. The mean difference is 1.00 with standard error of 1.155 (95% CI: − 3.2978 to 1.2978) with a poor correlation (correlation coefficient < 0.3). There was no significant difference in 6-month OKS between groups (*p* = 0.326) (Table 2).

**Table 2:** Operative and postoperative outcomes

Outcomes	Overall (*n* = 85)	No pain (*n* = 65)	Pain (*n* = 20)	Mean difference (95% CI)	*p*-value
Depth of tibial resection (mm)					
Anterior	7.3 ± 1.9	7.4 ± 1.8	7.0 ± 2.3	0.4 (− 0.6 to 1.4)	0.404
Middle	4.9 ± 1.8	4.8 ± 1.7	5.1 ± 2.1	− 0.2 (− 1.2 to 0.7)	0.611
Posterior	5.1 ± 1.7	5.0 ± 1.6	5.3 ± 2.0	− 0.4 (− 1.2 to 0.5)	0.372
Maximal depth	7.5 ± 1.9	7.5 ± 2.0	7.5 ± 1.4	0.1 (− 0.9 to 1.0)	0.909
Femoral component size					
Small	67 (78.8%)	50 (76.9%)	17 (85.0%)	NA	0.131
Medium	10 (11.8%)	10 (15.4%)	0 (0.0%)	NA	
Large	8 (9.4%)	5 (7.7%)	3 (15.0%)	NA	
Tibial component size					
AA	13 (15.3%)	10 (15.4%)	3 (15.0%)	NA	0.037
A	21 (24.7%)	18 (27.7%)	3 (15.0%)	NA	
B	28 (32.9%)	17 (26.2%)	11 (55.0%)	NA	
C	17 (20.0%)	16 (24.6%)	1 (5.0%)	NA	
D	3 (3.5%)	3 (4.6%)	0 (0.0%)	NA	
E	3 (3.5%)	1 (1.5%)	2 (10.0%)	NA	
Polyethylene thickness					
3 mm	48 (56.5%)	38 (58.5%)	10 (50.0%)	NA	0.825
4 mm	26 (30.6%)	19 (29.2%)	7 (35.0%)	NA	
5 mm	10 (11.8%)	7 (10.8%)	3 (15.0%)	NA	
6 mm	1 (1.2%)	1 (1.5%)	0 (0.0%)	NA	
Mean thickness	3.6 ± 0.7	3.6 ± 0.8	3.7 ± 0.7	−0.1 (−0.5 to 0.3)	0.617
Postoperative FTA (°)	179.8 ± 2.4	179.8 ± 2.3	179.8 ± 2.8	0.0 (1.2 to 1.2)	1.000
Tibial tray inclination (°)	88.5 ± 2.2	88.6 ± 2.2	88.2 ± 2.0	0.3 (−1.4 to 0.8)	0.589
Tibial tray slope (°)	4.2 ± 2.0	4.1 ± 2.0	4.3 ± 2.1	− 0.1 (− 1.1 to 0.9)	0.827
OKS (score)					
At 6 wk	32.6 ± 7.6	33.7 ± 6.5	28.9 ± 9.7	4.8 (0.0 to 9.6)	0.049
At 6 mon	40.8 ± 4.5	41.0 ± 4.8	40.0 ± 3.4	1.1 (− 1.2 to 3.5)	0.326

*CI* confidence interval, *FTA* mechanical femorotibial angle, *OKS* Oxford knee score, *NA* not applicable

For the analysis of factors that could significantly influence medial knee pain, variables with *p*-value < 0.2 (BMI, preoperative OKS, femoral and tibial component sizes) and depths of tibial resection were included in a logistic regression model. The univariate or multivariate analysis results revealed no independent association between an evaluated factor and medial knee pain (all *p* > 0.05).

## Discussion

This study’s cohort characteristics are equivalent to the recent largest OUKA cohort study in Thailand [16]. Our study provides solid evidence that tibial resection thickness in OUKA is not related to postoperative pain. In our study, the incidence of medial knee pain was 23.5%, and the P group had a lower OKS than the NP group. In stark contrast, Edmondson, et al. [6] reported a 55% incidence of medial knee pain after OUKA. Patients reporting medial knee pain in their study also had a poorer OKS. However, their study had a retrospective design with small sample size. In addition, they identified pain by inquiring that patients identify the region of greatest pain without doing a physical examination. Their rate may have been higher due to the difficulty in distinguishing medial knee pain from other types of knee pain and surrounding soft tissue pathologies. Ruangsomboon et al. conducted a physical examination of cutaneous pain and numbness 3 months after TKA and discovered that approximately 45% of patients experienced cutaneous pain [17]. Despite this, it is conceivable because the incision length and exposure of OUKA are often less and less invasive than those of TKA. In addition, unlike our investigation, theirs did not designate a specific medial location to measure. Therefore, our investigation may discover less incidence of medial knee pain. Numerous orthopedists have explored and analyzed this postoperative pain issue, including sought out novel pain management for OUKA [18, 19]. In the present study, all patients experienced spontaneous resolution of medial knee pain 6 months following surgery; the bone remodeling process may explain this phenomena. After resolution of medial knee pain, the functional outcome also improved.

Our study was cautious of specific areas that defined medial knee pain and differentiated soft tissue pain from underlying bone pain. Although our method does not ensure absolute differentiation between these two types of pain, we are confident that the various criteria we used facilitated accurate medial knee pain identification. Our study’s prospective design also allowed us to exclude other possible causes, such as medial overhanging of components and cementing error.

Another important finding in this study was that we could not identify any factors independently associated with medial knee pain, including depth of tibia resection. Previous finite element analysis found tibial strain to be increased by 20% after UKA [7]. This excessive strain might stimulate nociceptors and cause pain [20]. According to the previous study in the TKA model [13], a 15 mm tibial resection increased strain up to 281% compared to a 5 mm standard resection. We hypothesized that this phenomenon might also occur in the UKA model, thus resulting in medial knee pain. However, our study could not prove this theory. This is because OUKA was performed using a minor bone cut than TKA, and the difference in tibial depth had a slight change of strain that did not affect the clinical symptoms.

Moreover, we found no significant correlation between component alignment and medial knee pain. Since the spherical femoral component of the OUKA was tolerant to malalignment up to 10 degrees [21] and Oxford partial knee micro-plasty instrumentation was proven to improve the accuracy of femoral component alignment [22], our study focused on tibial component alignment.

Tibial component coronal alignment or tibial inclination were the essential factors most often mentioned in several studies [8, 23]. The Oxford Knee Group recommends acceptable tibial malalignment of 5 degrees [23]. Zhu, et al. [8] analyzed the influence of tibial malalignment on change in stress and strain in OUKA, and recommended an optimal range of 4 degrees valgus to 4 degrees varus position.

## Limitations

This research has limitations. First, despite including 85 knees in our final analysis and the smallest number predicted in our sample size calculation, it is possible that our study lacked the power to detect statistically significant differences and associations between groups. Second, because we only considered OA patients, it is possible that our findings do not apply to patients with osteonecrosis. Thirdly, preoperative MRI bone marrow was related to lower pain levels following UKA [24], although we did not investigate this issue. Third, due to a paucity of MRI data, some individuals with ACL insufficiency and/or lateral compartment injury were later omitted. Fourth, only prostheses that were cemented were used. Two studies, none of which focused on medial knee pain, reported no difference between cemented and cementless prostheses in terms of clinical outcomes [23, 25]. We mainly focused on short-term outcomes, but some medial knee pain case necessitates a longer investigation. Lastly, our research meticulously distinguished medial knee pain from soft tissue and bone pain, which may be challenging to demonstrate in a clinical context. However, we have endeavored to use the numerous criteria we applied to facilitate the resolution of these challenges.

## Conclusion

Medial knee pain was found to be common in the early postoperative period after OUKA, but this pain spontaneously resolved by 6 months. As a range of tibial resection level, post-operative pain is not associated with tibial resection thickness in this study.

## Data Availability

The datasets generated and/or analyzed during the current study are not publicly available. These datasets were stored in our internal high-security level hard drive but are available from the corresponding author on reasonable request. Requests for data not shown in the body of this manuscript can be made to the corresponding author.
